# Heterologous expression of family 10 xylanases from *Acidothermus cellulolyticus* enhances the exoproteome of *Caldicellulosiruptor bescii* and growth on xylan substrates

**DOI:** 10.1186/s13068-016-0588-9

**Published:** 2016-08-22

**Authors:** Sun-Ki Kim, Daehwan Chung, Michael E. Himmel, Yannick J. Bomble, Janet Westpheling

**Affiliations:** 1 Department of Genetics, University of Georgia, Athens, GA USA; 2 Biosciences Center, National Renewable Energy Laboratory, Golden, CO USA; 3 The BioEnergy Science Center, Oak Ridge National Laboratory, Oak Ridge, TN USA

**Keywords:** Consolidated bioprocessing, Biomass deconstruction, Xylanase, *Caldicellulosiruptor*

## Abstract

**Background:**

The ability to deconstruct plant biomass without conventional pretreatment has made members of the genus *Caldicellulosiruptor* the target of investigation for the consolidated processing of lignocellulosic biomass to biofuels and bioproducts. These Gram-positive bacteria are hyperthermophilic anaerobes and the most thermophilic cellulolytic organisms so far described. They use both C5 and C6 sugars simultaneously and have the ability to grow well on xylan, a major component of plant cell walls. This is an important advantage for their use to efficiently convert biomass at yields sufficient for an industrial process. For commodity chemicals, yield from substrate is perhaps the most important economic factor. In an attempt to improve even further the ability of *C. bescii* to use xylan, we introduced two xylanases from *Acidothermus cellulolyticus*. Acel_0180 includes tandem carbohydrate-binding modules (CBM2 and CBM3) located at the C-terminus, one of which, CBM2, is not present in *C. bescii*. Also, the sequences of Xyn10A and Acel_0180 have very little homology with the GH10 domains present in *C. bescii*. For these reasons, we selected these xylanases as potential candidates for synergistic interaction with those in the *C. bescii* exoproteome.

**Results:**

Heterologous expression of two xylanases from *Acidothermus cellulolyticus* in *Caldicellulosiruptor bescii* resulted in a modest, but significant increase in the activity of the exoproteome of *C. bescii* on xylan substrates. Even though the increase in extracellular activity was modest, the ability of *C. bescii* to grow on these substrates was dramatically improved suggesting that the xylan substrate/microbe interaction substantially increased deconstruction over the secreted free enzymes alone.

**Conclusions:**

We anticipate that the ability to efficiently use xylan, a major component of plant cell walls for conversion of plant biomass to products of interest, will allow the conversion of renewable, sustainable, and inexpensive plant feedstocks to products at high yields.

**Electronic supplementary material:**

The online version of this article (doi:10.1186/s13068-016-0588-9) contains supplementary material, which is available to authorized users.

## Background

Members of the genus *Caldicellulosiruptor* have the unusual ability to grow on a variety of lignocellulosic biomass substrates without conventional pretreatment [1]. Unlike many of the cellulolytic clostridia that rely on complex protein structures called cellulosomes to attach to and solubilize plant cell walls, *Caldicellulosiruptor* species secrete primarily free multifunctional enzymes into the exoproteome. We use the term exoproteome instead of secretome, originally coined by Antelmann et al. [2] and further defined by Tjalsma et al. [3], to refer to both the secreted proteins and the process of secretion. Thus, the term secretome of a microorganism provides an integrated and global view of its secretion pathways and routing of the secreted proteins, as well as their post-translational modifications, their transport and their final subcellular localization. Some secreted proteins are released into the extracellular environment, whereas others remain anchored to the membrane or cell wall [4]. The exoproteome refers to only one aspect of the secretome [5], the subset of proteins present in the extracellular milieu (supernatant for the purposes of this work). Species of *Caldicellulosiruptor* vary in their ability to deconstruct plant biomass, and the degree to which they are able to utilize biomass substrates for growth varies with the composition and activity of several specific enzymes they produce. The *C. bescii* genome encodes 52 glycoside hydrolases, many of which are multimodular and multifunctional, including several endo- and exocellulases. The most prominent among them is CelA [6, 7]. It also contains a xylan-degrading enzyme mixture consisting of xylanases (Cbes_0183 and Cbes_0185), β-d-xylosidase (Cbes_2354), α-l-arabinofuranosidase (Cbes_1103), α-d-glucuronidase (Cbes_0854), and acetylxylan esterase (Cbes_0152) [8]. Of the six putative xylanases encoded in the *C. bescii* genome, only one is multifunctional (Cbes_1857), two others contain CBM22s that are known to bind xylan with a thermostabilizing effect, two contain a single GH10, and one contains a GH11 with a CBM36 (Table 1). *C. bescii* exhibits significant xylanase activity and grows well on both oat spelts and birchwood xylans. It can simultaneously use xylans and glucans and actually grow better on xylan [1, 6].

**Table 1:** List of glycoside hydrolase Family 10 (GH10) catalytic domains in *Caldicellulosiruptor bescii* and their sequence homology with the GH10 domains in Acel_0180 and Acel_0372 xylanases from *A. cellulolyticus*

ORF	CAZy module (architecture/size)	Protein sequence homology with Acel_0180 (coverage/identity)	Protein sequence homology with Acel_0372 (coverage/identity)
Cbes_2724	GH10 (1.224 kb)	85 %/28 %	92 %/27 %
Cbes_0183	CBM22-CBM22-GH10 (2.091 kb)	96 %/35 %	97 %/35 %
Cbes_0185	GH10 (0.981 kb)	98 %/31 %	95 %/29 %
Cbes_1857	GH10-CBM3-CBM3-GH48 (3.434 kb)	96 %/36 %	97 %/32 %
Cbes_0618	CBM22-CBM22-GH10 (2.064 kb)	98 %/33 %	96 %/31 %

Other somewhat similar bacteria such as the Gram-positive thermophilic actinomycete *Acidothermus cellulolyticus* contain CAZymes with much simpler architectures that exhibit high specific activity on biomass at elevated temperatures [9]. *Acidothermus cellulolyticus* in particular produces many CAZymes that might act synergistically with the *C. bescii* exoproteome. One such example is the cellulase E1, a thermostable, endo-1,4-β-glucanase (GH5) with a family 2 carbohydrate-binding module. We recently reported the expression and secretion of the E1 protein from *A. cellulolyticus* in *C. bescii* and showed that its presence increased the specific activity of the *C. bescii* exoproteome on biomass [10], presumably acting in concert with the most efficient and most highly expressed cellulase in *C. bescii,* CelA [7].

The term, xylan, describes polysaccharides that have β-(1,4)-D-xylopyranose backbones with a variety of side chains usually attached at the *O*-2 and *O*-3 positions and include glucuronic acid, 4-*O*-methylglucuronic acid, l-arabinofuranose, xylose, and acetyl groups. The types and levels of side chains are dependent on the particular plant source, with hardwoods having high acetyl and glucuronic acid moieties (glucuronoxylans) and grasses having mainly arabinofuranose and acetyl groups (arabinoxylan) [11]. Because of the complex nature of xylans, their enzymatic hydrolysis is intrinsically more complicated than that of most other plant polysaccharides. In contrast to cellulases, hemicellulases include a much broader range of activities. In addition to analogous versions of endo-, exo-, and glycosidase cellulase activities, multiple debranching activities are also required for efficient growth on biomass. The varied backbone composition of hemicelluloses also adds to this complexity, where xylans, xyloglucans, mannans, and numerous other minor polysaccharide chains are associated with different hemicelluloses.

Xylanases, in general, fall in the two glycoside hydrolase families, GH10 and GH11. In addition, there are reports of xylanase activity exhibited by other glycoside hydrolase families such as GH5, GH8, and GH43 [12]. These xylan-degrading GHs are associated with several families of carbohydrate-binding modules (CBMs) including families 2, 3, 4, 6, 22, and 36. Xylanases range from those containing a single catalytic domain to large multimodular proteins such as those commonly found in *C. bescii* or part of even larger molecular assemblies such as the cellulosome [13]. Xyn10A (Acel_0372) is a thermostable family 10 glycoside hydrolase (GH10) from the Gram-positive thermophilic actinomycete, *Acidothermus cellulolyticus,* that does not contain a CBM. It is classified as an endoxylanase with optimal activity at pH 6 and a temperature range of activity to 90 °C [14]. Its activity is, in fact, stabilized against thermal denaturation in the presence of the substrate [14]. Transcription of the gene for Xyn10A is upregulated during growth on xylan substrates and the enzyme is most active on birchwood xylan [14]. Acel_0180 is the only other xylanase in the *A. cellulolyticus* genome containing a GH10 catalytic domain. This protein includes a tandem of carbohydrate-binding modules (CBM2 and CBM3) located at the C-terminus. While nothing is known or published about this xylanase, its architecture differs significantly from those found in *C. bescii.* Because these xylanases are more simple in structure and different from those found in *C. bescii*, both Xyl10A and the putative xylanase encoded by Acel_0180 represent potential candidates for synergy studies with the *C. bescii* exoproteome.

Here, we show that expression of either of these xylanases had a modest but significant effect on the activity of the *C. bescii* exoproteome, but a dramatic effect on the ability of *C. bescii* to grow on xylan substrates.

## Results and discussion

### Heterologous expression and secretion of two xylanases from *A. cellulolyticus* 11B in *C. bescii*

**Fig. 1:**
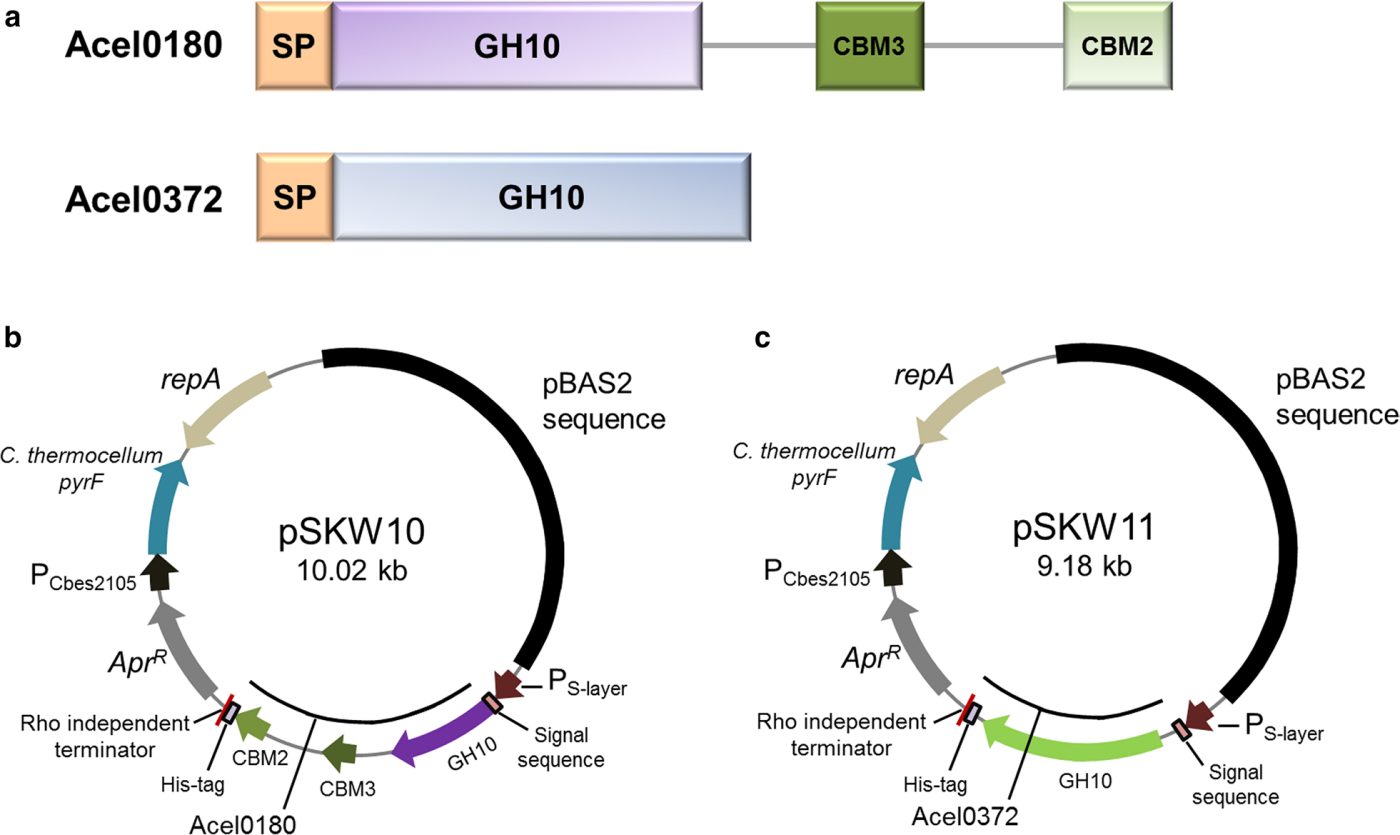
Construction of vectors for the expression of *A. cellulolyticus* xylanases in *C. bescii*. **a** Schematic diagrams of Acel_0180 and Acel_0372 proteins: SP, signal peptide; GH10, a family 10 glycoside hydrolase; CBM3, a family 3 carbohydrate-binding module; CBM2, a family 2 carbohydrate-binding module. Maps of expression vectors for Acel_0180 (**b**) and Acel_0372 (**c**) expression. Genes were expressed under the transcriptional control of the *C. bescii* S-layer promoter. The expression vectors contain a CelA signal sequence, a C-terminal 6X his tag, a rho-independent terminator, the *pyrF* (from *C. thermocellum*) cassette for selection, and pBAS2 sequences for replication in *C. bescii*

To construct expression plasmids for the Acel_0180 and Acel_0372 xylanases from *A. cellulolyticus* (Fig. 1a) in *C. bescii*, the genes were amplified from *A. cellulolyticus* gDNA and cloned into shuttle vectors, pSKW10 and pSKW11 (Fig. 1b, c), under the transcriptional control of the *C. bescii* S-layer promoter [15, 16] containing the CelA signal sequence for protein secretion. CelA is the most abundant extracellular protein produced by *C. bescii* [1, 17] and we recently reported the use of this signal peptide for the secretion of E1 in *C. bescii* [10]. The recipient strains also contained the *A. cellulolyticus* E1 gene inserted into the *C. bescii* chromosome as a chromosomal insertion site (CIS1) (Table 2). Insertion of DNA at this site was determined not to affect growth and has no detectable effect on the cell [10]. The construction of this strain and its comparison to wild-type *C. bescii* has been recently published [10]. The CelA signal sequence was attached to the N-terminus of each protein replacing the native signal peptides. pSKW10 or pSKW11 also contain a wild-type *pyrF* allele from *Clostridium thermocellum* and were transformed into JWCB52 that contains a *pyrF* deletion resulting in uracil auxotrophy [10]. Transformants were selected by uracil prototrophy to generate JWCB74 (containing pSKW10) and JWCB75 (containing pSKW11). The resulting strains were grown at 65 °C to accommodate the efficient expression of *C. thermocellum pyrF* gene on the plasmid, and PCR analysis was used to confirm the presence of the plasmids. Primers (DC460 and DC228) were used to amplify the portion of the plasmid containing the open reading frame of the targeted proteins, but also annealing to regions of the plasmid outside the gene to avoid amplification of sequences residing on the chromosome (Additional file 1: Fig. S1A). Total DNA from these strains was used to back-transform *E. coli*, and two different restriction endonuclease digests performed on plasmid DNA purified from three independent back-transformants of *E. coli* resulted in identical digestion patterns that were the same as the original plasmid (Additional file 1: Fig. S1B, C). These results show that the plasmids were successfully transformed into *C. bescii* and were structurally stable during transformation and replication in *C. bescii* and back-transformation to *E. coli.*

**Table 2:** Strains and plasmids used in this study

Name	Description	Reference
*E. coli*		
JW520	DH5α containing pSKW010 (Apramycin^R^)	This study
JW521	DH5α containing pSKW011 (Apramycin^R^)	This study
*C. bescii*		
JWCB18	*ΔpyrFA ldh::ISCbe4 Δcbe1*(*ura* ^−^ */*5-FOA^R^)	[40]
JWCB52	*ΔpyrFA ldh::ISCbe4 Δcbe1 CIS1::P*_S-layer_ *acel0614(E1)* (*ura* ^−^ */*5-FOA^R^)	[10]
JWCB21	JWCB18 containing pDCW89 (*ura* ^+^ */*5-FOA^S^)	[43]
JWCB73	JWCB52 containing pJGW07 (*ura* ^+^ */*5-FOA^S^)	This study
JWCB74	JWCB52 containing pSKW10 (*ura* ^+^ */*5-FOA^S^)	This study
JWCB75	JWCB52 containing pSKW11 (*ura* ^+^ */*5-FOA^S^)	This study
Plasmids		
pDCW89	*E. coli*/*C. bescii* shuttle vector containing the *C. bescii pyrF* gene (Apramycin^R^)	[43]
pJGW07	*E. coli*/*C. bescii* shuttle vector containing the *C. thermocellum pyrF* gene (Apramycin^R^)	[41]
pDCW173	Intermediate vector 1 (Apramycin^R^)	[16]
pDCW212	Intermediate vector 2 (Apramycin^R^)	This study
pDCW213	Source of Acel_0180 open reading frame (Apramycin^R^)	This study
pDCW214	Source of Acel_0372 open reading frame (Apramycin^R^)	This study
pSKW10	Expression vector containing *P*_S-layer_—Acel_0180 (Apramycin^R^)	This study
pSKW11	Expression vector containing *P*_S-layer_—Acel_0372 (Apramycin^R^)	This study

**Fig. 2:**
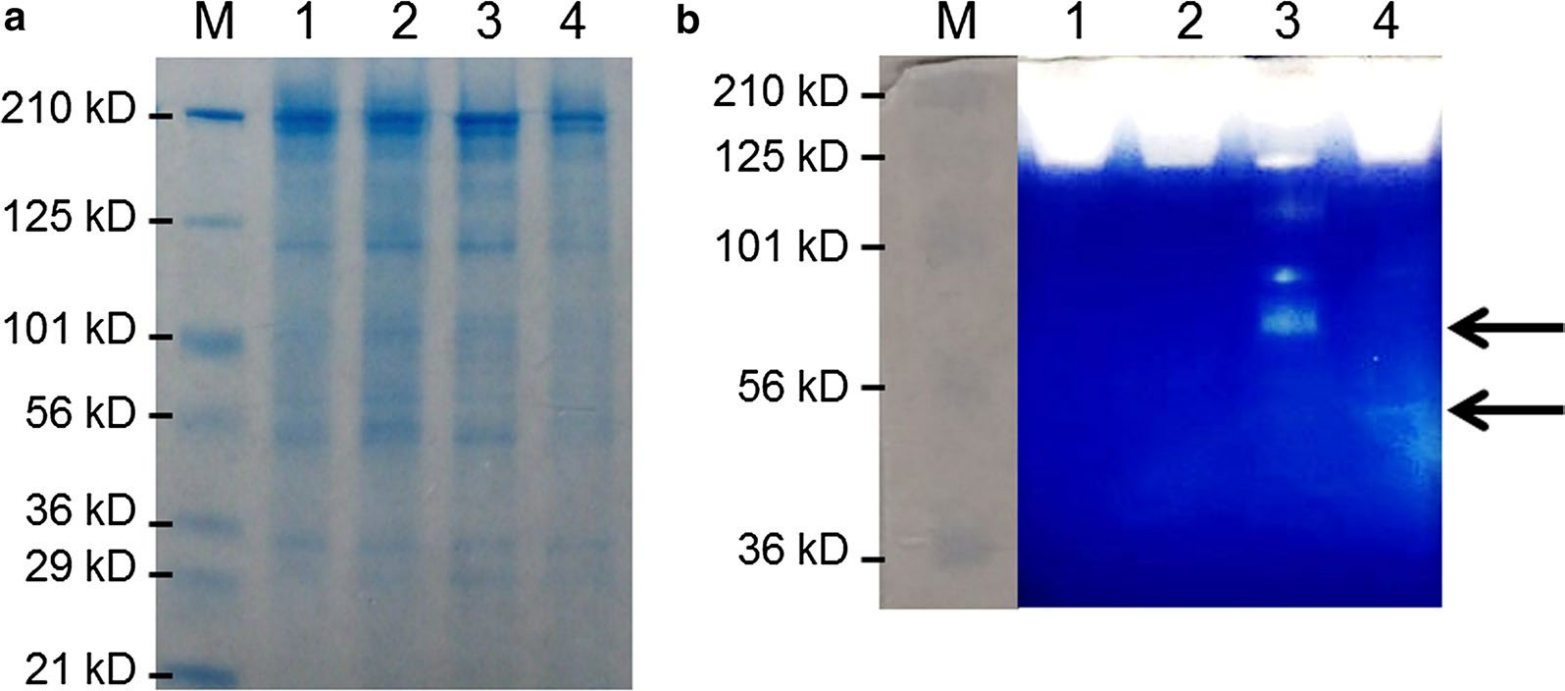
Confirmation of xylanase expression and activity in *C. bescii*. **a** SDS-PAGE analysis of concentrated extracellular proteins (20 µg). **b** Zymogram analysis of concentrated extracellular proteins (4 µg). *M*, Pre-stained SDS-PAGE standards, broad range (Bio-Rad Laboratories); *1*, extracellular fraction from JWCB21 (parental strain); *2*, JWCB73 (E1 expressing strain); *3*, JWCB74 (E1 and Acel_0180 expressing strain); *4*, JWCB75 (E1 and Acel_0372 expressing strain)

To examine the expression and secretion of the Acel_0180 and Acel_0372 xylanases in *C. bescii*, strains JWCB74 and JWCB75 (Table 2) were grown in low osmolarity defined (LOD) medium with 40 mM MOPS [18]. The cells were removed by centrifugation and filtration and the remaining extracellular fraction was concentrated (1000-fold) and displayed on a polyacrylamide gel containing 0.1 % birchwood xylan. Although the xylanase proteins were difficult to visualize using Coomassie blue staining (Fig. 2a), their activity was clearly detected by zymogram analysis using birchwood xylan as a substrate with Congo red staining (Fig. 2b). The background color of the zymogram turned blue after submerging the gel in a 5 % acetic acid solution. These results show that the Acel_0180 and Acel_0372 xylanases were expressed and functional in *C. bescii* and suggest that they were secreted to the exoproteome. Comparison of the band intensities from the zymogram showed that the xylanase activity band of Acel_0180 (69 kDa) was more than ten times greater than that of the Acel_0372 xylanase (42 KDa). One possible explanation for this is the presence of a CBM2 at the C-terminal end of Acel_0180 (Fig. 1a) that would be expected to increase binding of the enzyme to the xylan substrate. Even though this CBM is of type 2a (Additional file 1: Fig. S2), known to be more specific to cellulose, it still has affinity for insoluble xylan [19]. In the Acel_0180 xylanase expression strain, a xylanase activity band with a higher molecular weight (MW) than that predicted for Acel_0180 (69 kDa) was also detected. We speculate that the difference from the predicted molecular weight might be due to in vivo glycosylation of Acel_0180 xylanase in *C. bescii.* The linkers between the two carbohydrate-binding domains of Acel_0180 are serine rich and have been identified as potential sites for O-glycosylation. We, in fact, recently reported that CelA is glycosylated when produced natively in *C. bescii* [16].

### Expression of the xylanases in *C. bescii* results in an increase in the activity of the exoproteome on xylan

**Fig. 3:**
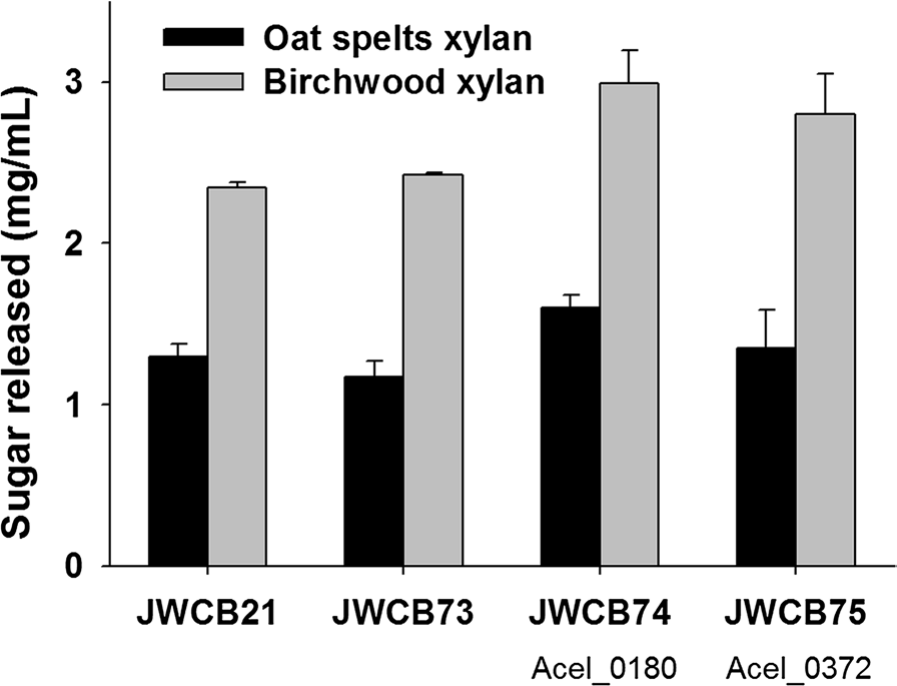
Relative enzymatic activity of the extracellular fraction of *C. bescii* strains on oat spelts and birchwood xylans. Activity of extracellular protein (25 µg/mL concentrated protein) on oat spelts and birchwood xylans was measured after 12 h incubation at 75 °C in triplicate. JWCB21, the parent strain used in these experiments (see Table 2 for genotype details); JWCB73, the E1 expression strain; JWCB74, the E1 expression strain containing Acel_0180; JWCB75, the E1 expression strain containing Acel_0372

To examine whether expression of the Acel_0180 and Acel_0372 xylanases in *C. bescii* enhanced the hemicellulolytic activity of the exoproteome, enzyme assays were performed using oat spelts and birchwood xylans as substrates. Oat spelts xylan is a complex arabinoxylan, branched with arabinose residues. Birchwood xylan is a simpler, primarily unsubstituted xylose polymer with traces of uronic acids as side groups (more than 90 % β-1,4-linked xylose residues) [20, 21]. It is important to note that both xylan preparations have approximately 2–3 % residual glucose moieties within the xylan structure [22]. The extracellular protein fraction from JWCB21 (the parental strain), JWCB73 (the parental strain containing E1), JWCB74 containing the Acel_0180 xylanase, and JWCB75 containing the Acel_0372 xylanase were compared (Fig. 3). Cells were grown at 65 °C and supernatants were concentrated 1000-fold and exchanged with 20 mM MES/2 mM β–mercaptoethanol (pH 5.5) buffer before assaying on oat spelts and birchwood xylans for 1 and 12 h at 65 and 75 °C. Thermostability and activity of *C. bescii* xylan-degrading enzymes are known to decrease significantly at temperatures higher than 85 °C. Although *A. cellulolyticus* cells grow optimally at 55 °C [23], the optimal temperature for in vitro xylanase activity of Acel_0372 was reported to be 90 °C [14] which is even higher than that of E1 of 80 °C [24].

The xylanase activity of the parental strain (JWCB21) and the E1 expressing strain (JWCB73) were found to be almost identical (Fig. 3; Additional file 1: Fig. S3, S4). While the activity of the exoproteome from the Acel_0372 expressing strain (JWCB75) on oat spelts xylan was indistinguishable from that of the control strains, the activity of this exoproteome on birchwood xylan increased 19 % after 12 h incubation at 75 °C (*P*_value_ = 0.042). This is consistent with previous studies showing that the activity of Acel_0372 was higher on birchwood xylan than on oat spelts xylan [14]. After 12 h incubation at 75 °C, culture supernatants from the Acel_0180 expression strain (JWCB74) showed 23 % (*P*_value_ = 0.005) and 27 % (*P*_value_ = 0.015) higher xylanase activity on oat spelts and birchwood xylans, respectively. The extent of improvement was more significant after 1 h incubation at 75 °C (Additional file 1: Fig. S3), and, while total xylanase activity decreased at 65 °C, the overall activity increase observed in strains expressing Acel_0180 was constant at this temperature (Additional file 1: Fig. S4). These results suggest that both xylanases enhance the exoproteome activity of *C. bescii* to approximately the same extent in spite of the fact that the exoproteome already contains high levels of xylanase activity [8]. The *A. cellulolyticus* xylanases were produced from a constitutive promoter not dependent on regulation by the substrate. The low molecular weight xylanases from *C. bescii* are very low in abundance, if detected at all, in proteomics analyses of the exoproteome—with the exception of the GH11 containing Cbes_0089, which is expressed at a level 30-fold below that of CelA [17].

The architecture of these xylanases may also play a role in their activity. For example, the large multifunctional xylanases containing a GH10 in *C. bescii* may get trapped in non-productive binding on these substrates and reduce their activity. It is also possible that these heterologous xylanases have a higher specific activity given that they have a poor homology with those already present in the *C. bescii* exoproteome (Table 1). Acel_0372 was previously reported to have high activity on both substrates tested in this study [14]. The presence of the CBM modules may also affect activity. Even though the CBM2 of Acel_0180 is not predicted to bind xylan strongly since it is of Type A [25], there most likely exists more binding capacity than there is for a single catalytic domain (Additional file 1: Fig. S2), explaining the increase over Acel_0372. Additionally, due to the residual glucose moieties within the xylan structure in both substrates, the CBM2 and CBM3 modules (type b, see Additional file 1: Fig. S5; [26]) may target these sites and digest nearby xylan. The differences observed between the two GH10s in Acel_0372 and Acel_0180 is most likely not due to their intrinsic level of activity. As shown in Additional file 1: Fig. S6, S7, there is nothing obvious in their predicted structures that would indicate better activity from the catalytic domain of Acel_0180 over Acel_0372. The catalytic residues are identical and the binding pockets are very similar. Of course, other factors could change the activity of each GH10 such as changes in pKas due to the environment of the catalytic site, but given the low homology of the templates used to build the homology models it is not tractable without more reliable structures beyond the scope of this study.

### Expression of the *A. cellulolyticus* xylanases in *C. bescii* results in a dramatic increase in its ability to grow on xylan substrates

**Fig. 4:**
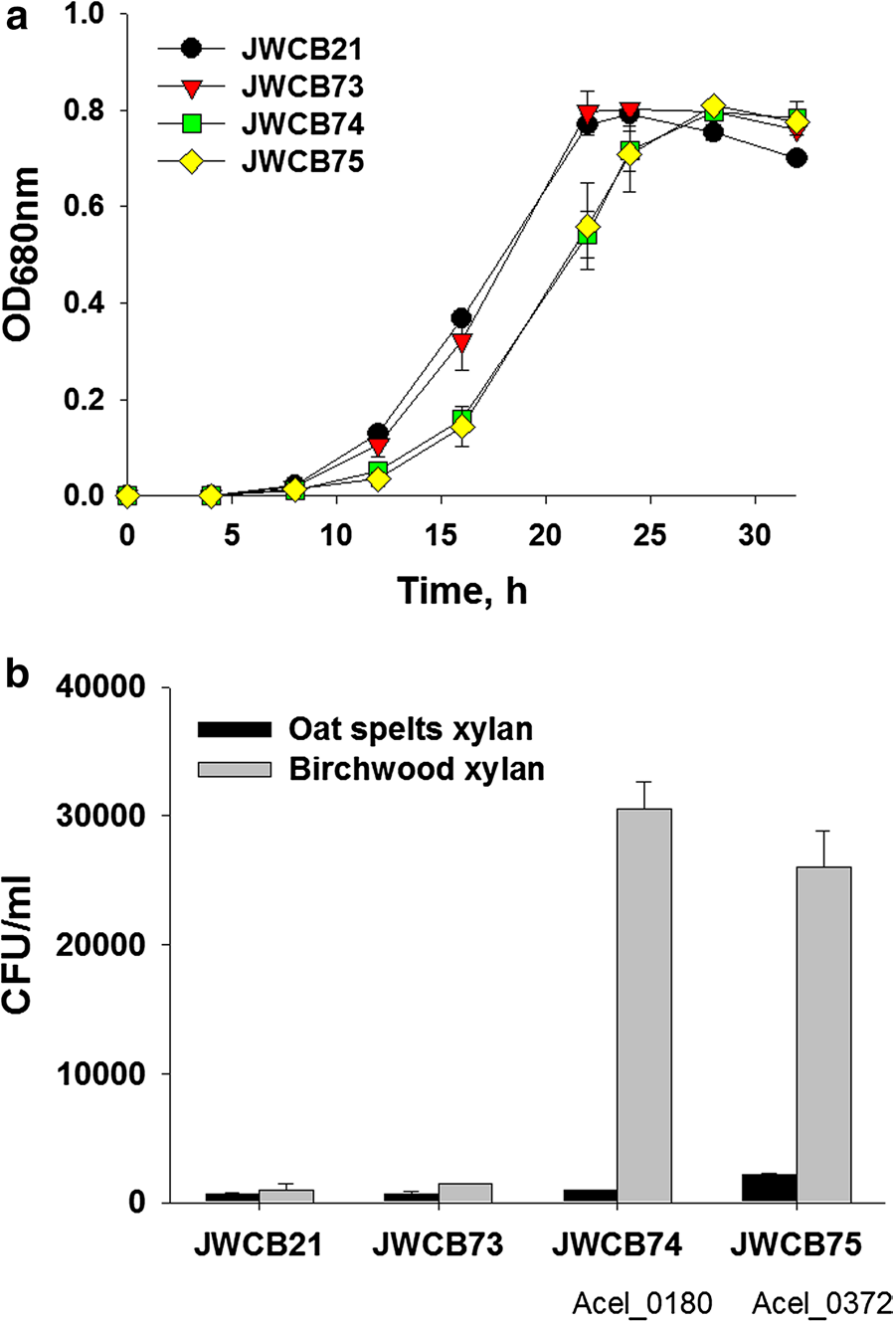
Growth of *C. bescii* strains on cellobiose (**a**) or xylan substrates (**b**). JWCB21, the parent strain used in these experiments (see Table 2 for genotype details); JWCB73, the E1 expression strain; JWCB74, the E1 expression strain containing Acel_0180; JWCB75, the E1 expression strain containing Acel_0372. Viable cell counts are measured after 36 h cultivation on xylan substrates

Both the Acel_0180 and Acel_0372 xylanases contain GH10 catalytic domains (Fig. 1a). Family GH10 xylanases show both endo-β-1,4-xylanase and endo-β-1,3-xylanase activity, but very little activity on either cellulose and cellobiose [27]. Growth was first measured on the soluble substrate, cellobiose, as there should be no effect on growth with this substrate. As expected, growth of the parent strain, the E1 expressing strain, and the strains containing the heterologous xylanases (JWCB74 and JWCB75) was virtually identical (Fig. 4a). While there was a slightly longer lag phase in the growth of JWCB74 and JWCB75, the final cell density was almost identical. These data also show that expression of these xylanases in *C. bescii* had no obvious detrimental effect on growth in general. We next examined the effect of expression of these xylanases on the growth of *C. bescii* on xylan. *C. bescii* grows well on xylan as sole carbon source, to approximately 5 × 10^9^ cells/ml after 24 h using cell counts as measured by fluorescence staining using acridine orange [28]. Because xylan is an insoluble substrate, this type of assay is essential for accurate measurements of growth as it eliminates error from counting particulate plant biomass as the biomass does not fluoresce. Another, perhaps even more accurate and less labor-intensive assay is to measure the number of viable cells after growth on insoluble substrates. The advantage of this method is that only cells will be counted eliminating errors due to biomass, but the disadvantage of using viable cell counts is that it underestimates the total number of cells. We previously reported plating efficiencies for *C. bescii* on selective solid medium [29]. The number of viable cells is approximately 1000-fold lower than cell counts using fluorescence and we emphasize that both methods report relative cell numbers. Viable cell counts after 12, 24, and 36 h cultivation on oat spelts and birchwood xylans are reported (Fig. 4b; Additional file 1: Fig. S8). Viable cell counts on oat spelts xylan were higher than on birchwood xylan after 12 h, but growth on birchwood xylan increased significantly over time. This is consistent with the observed xylanase activity (Fig. 3; Additional file 1: Fig. S3, S4), indicating early saturation of xylanase activity on oat spelts xylan, likely because of its highly branched structure. As shown in Fig. 4b; Additional file 1: Fig. S8, the expression of either xylanase in *C. bescii* resulted in an increase in the ability of these strains to grow on xylan substrates. The parental (JWCB21) and E1-expressing (JWCB73) strains grew well on this substrate and the histogram is a bit misleading, because the difference between the strains is dramatic. After 36 h cultivation on oat spelts xylan (6.5 × 10^2^), the xylanase-expressing strains (JWCB74 and JWCB75) showed 1.0 × 10^3^ and 2.2 × 10^3^ CFU/mL cell densities, respectively. This growth phenotype of xylanase-expressing strains was also seen on birchwood xylan. Growth of the JWCB74 and JWCB75 on birchwood xylan was 30.5- (*P*_value_ = 0.013) and 26.0-fold (*P*_value_ = 0.023) higher than that of the parental strain (Fig. 4b; Additional file 1: Fig. S9). Growth of JWCB73 expressing the E1 protein from *A. cellulolyticus* on birchwood xylan was also enhanced by 50 % compared with that of the JWCB21. E1 is a GH5 family endo-β-1,4-glucanase, and a previous study reported that a similar endo-β-1,4-glucanase of the GH5 family from *Sporotrichum thermophile* (StCel5A) was active on birchwood xylan [30]. Based on this report, we anticipated that the E1 endoglucanase would be active on birchwood xylan and this was shown to be true. Interestingly, the effect of these xylanases appears far greater on growth than on the activity of the exoproteomes. We suggest that the difference between the activity of the free enzymes and growth of the organism containing these enzymes is due to an increase in the local concentration of enzymes secreted, as cells are transiently attached to the substrate.

## Conclusions

Xylose is one of the most abundant sugars in lignocellulosic biomass [31]. Most industrial microorganisms that quantitatively convert glucose to ethanol, including *Saccharomyces cerevisiae* [32–34], *Clostridium thermocellum* [35, 36], and *Escherichia coli* [37, 38], however, cannot efficiently utilize C5 sugars. *C. bescii* grows well on cellulose, hemicellulose, and un-pretreated lignocellulosic biomass [35], and in fact grows better on xylan than crystalline cellulose [1]. This makes *C. bescii* a potential candidate for cost-competitive production of biofuels and biochemicals from lignocellulosic biomass with high xylan content.

## Methods

### Strains, media, and growth conditions

Strains of *E. coli* and *C. bescii* used in this study are listed in Table 2. *Caldicellulosiruptor* strains were grown under anaerobic conditions at 65 °C on solid or in liquid low osmolarity-defined (LOD) medium [39], as previously described, with maltose (0.5 % *w/v*) or cellobiose (0.5 %) as sole carbon source for routine growth and transformation experiments [40]. For growth of uracil auxotrophs, the defined medium contained 40 µM uracil. At this concentration, uracil does not support the growth of *C. bescii* as the sole carbon source. *E. coli* strain DH5α was used as a host for plasmid DNA construction and preparation using standard techniques. *E. coli* cells were cultured in LB broth containing apramycin (50 µg/mL). Plasmid DNA was isolated using a Qiagen Miniprep Kit (Qiagen, Valencia, CA, USA). Chromosomal DNA from *Caldicellulosiruptor* strains was extracted using the Quick-gDNA MiniPrep (Zymo, Irving, CA, USA) as previously described [29].

### Construction and transformation of xylanase expression vectors

Plasmids were constructed using Q5 High-Fidelity DNA polymerase (New England BioLabs, Ipswich, MA, USA) for PCR reactions, restriction enzymes (New England BioLabs, Ipwich, MA, USA), and the Fast-link DNA ligase kit (Epicentre Biotechnologies, Madison, WI, USA) according to the manufacturer’s instruction. Plasmid pSKW10 (Fig. 1b) was constructed in two cloning steps (Additional file 1: Fig. S10). First, a 1.9 kb DNA fragment containing the coding sequence of Acel_0180 without the signal sequence was amplified with primers DC701 (with an ApaLI site) and DC702 (with an AvrII site) using *A. cellulolyticus* 11B gDNA as template. The 5.9 kb DNA fragment containing the regulatory region of Cbes2303 (*P*_S-layer_), the signal CelA signal sequence, a C-terminal 6X histidine tag, and a rho-independent transcription terminator was amplified by PCR with primers DC699 (with ApaLI site) and DC700 (with AvrII site) using pDCW212 as template. This 5.9 kb DNA fragment was also amplified from plasmid pDCW175 [10], as plasmid pDCW212 is identical to pDCW175 except for the 5′ and 3′ flanking regions designed for future integration of target genes into the *C. bescii* chromosome (Additional file 1: Fig. S10). These two linear DNA fragments were digested with ApaLI and AvrII and ligated to construct a 7.8 kb intermediate vector, pDCW213. In a second step, the 2.1 kb Acel_0180 expression cassette, containing the regulatory region of Cbes2303 (S-layer protein), the CelA signal sequence, a C-terminal 6X histidine tag, and a rho-independent transcription terminator, was amplified by PCR with primers DC460 (with PvuI site) and DC461 (with NotI site) using pDCW213 as template. A 7.9 kb DNA fragment containing the pSC101 replication origin for *E. coli*, a *C. bescii* replication origin from pBAS2, an apramycin resistance gene cassette (Apr^R^), and a *C. thermocellum pyrF* expression cassette was amplified with primers DC481 (with PvuI site) and DC482 (with NotI site) using pDCW173 as template. These two linear DNA fragments were digested with Pvu1 and Not1 and ligated to construct pSKW10 (Fig. 1b). Plasmid pSKW11 is identical to pSKW10 except for the target protein sequence (Fig. 1). To make this change, plasmid pDCW214 was constructed by inserting the coding sequence of Acel_0372 without the signal sequence into pDCW212, which contains the regulator region of Cbes2303 (S-layer protein), the CelA signal sequence, a C-terminal 6X histidine tag, and a rho-independent transcription terminator. A 1.1 kb DNA fragment containing the coding sequence of Acel_0372 without the signal sequence was amplified with primers DC703 (with ApaLI site) and DC704 (with AvrII site) using *A. cellulolyticus* 11B gDNA as template. In the next step, the 1.3 kb Acel_0372 expression cassette was amplified with the primers DC560 (with BamHI site) and DC461 (with NotI site) using pDCW214 as template. A 7.9 kb DNA fragment containing the pSC101 replication origin for *E. coli*, a *C. bescii* replication origin, an apramycin resistance gene cassette (Apr^R^), and a *C. thermocellum pyrF* expression cassette was amplified with primers DC464 (with BamHI site) and DC482 (with NotI site) using pDCW173 as template. These two linear DNA fragments were digested with BamHI and NotI and ligated to construct pSKW11 (Fig. 1c). These plasmids were introduced into *E. coli* DH5α cells by electroporation in a 1-mm-gap cuvette at 1.8 kV and transformants were selected for apramycin resistance. All plasmids were sequenced by automatic sequencing (Genewiz, South Plainfield, NJ, USA). Electrotransformations in *C. bescii* cells were performed as previously described [41]. After being electro-pulsed with plasmid DNA (~0.5 μg), the cultures were recovered in low osmolarity complex (LOC) medium [39] at 75 °C. Recovery cultures were transferred into liquid LOD medium [39] without uracil to select uracil prototrophic plasmid transformants. Cultures were plated on solid LOD media to obtain isolated colonies, and total DNA was extracted. Taq polymerase (Sigma, St. Louis, MO, USA) was used for PCR reactions to confirm the presence of the plasmid. PCR amplification was done with primers (DC460 and DC228) outside of the gene cassette on the plasmid to confirm the presence of gene insertion. Primers used for plasmid constructions and confirmations are listed in Additional file 1: Table S1.

### Preparation of extracellular protein and zymogram analysis

To collect the extracellular protein (ECP) fraction, *C. bescii* cells were grown in 2 L of LOD medium with 40 mM MOPS containing 0.5 % cellobiose in closed bottles at 65 °C with shaking at 150 rpm to an OD_680_ of 0.25–0.3. The culture broth was centrifuged (6000×*g* at 4 °C for 15 min) and filtered (glass fiber, 0.7 µm) to separate out cells and concentrated with a 10 kDa molecular weight cutoff column. The concentrated ECP was exchanged with buffer 20 mM MES/2 mM β–mercaptoethanol (pH 5.5), and protein concentrations were determined using the Bio-Rad protein assay kit with bovine serum albumin (BSA) as the standard. ECP samples (20 µg) were electrophoresed in 4–15 % gradient Mini-Protein TGX gels (BIO-RAD) and protein bands were visualized by staining with Coomassie Brilliant Blue G-250. For the zymogram analysis, ECP samples (4 µg) were electrophoresed in 12 % polyacrylamide gel with a 5 % stacking gel containing 0.1 % birchwood xylan. The zymogram gel was soaked for 1 h in 2.5 % (*v/v*) Triton X-100 solution to remove the SDS and washed in distilled water. After incubating the gel at 75 °C for 30 min in the reaction buffer containing 20 mM MES (pH 5.5), 1 mM dithiothreitol (DTT), 1 mM CaCl_2_, and 1 mM MgCl_2_, the gel was submerged in 0.1 % (*w/v*) Congo red solution for 30 min and destained with 1 M NaCl until pale-red hydrolysis zones appeared. The reaction was stopped by dipping the gel into a 5 % acetic acid solution. The quantification of band intensity was carried out using the densitometry software (Total Lab 1.01, Nonlinear Dynamics Ltd.)

### Enzyme activity assays

Enzyme activity on xylan substrates was measured using 10 g/L of either oat spelts or birchwood xylan in MES reaction buffer (pH 5.5) as previously described [42]. Cells were grown in a 2 L volume of LOD medium with 40 mM MOPS and maltose as carbon source. 25 µg/mL of the extracellular protein fraction was added to each reaction and incubated at 65 and 75 °C for 1 and 12 h. Reducing sugars in the supernatant were measured using dinitrosalicylic acid (DNS). Samples and standards (xylose) were mixed 1:1 with DNS reaction solution, boiled for 2 min, and measured at *OD*_575_. Activity was reported as mg/mL of sugar released.

### Growth of recombinant strains on cellobiose and xylan

To measure growth on cellobiose, cells were sub-cultured twice in LOD medium with 5 g/L maltose as sole carbon source and this culture was used to inoculate media with cellobiose (1 % total volume for all experiments) as the sole carbon source at a final concentration of 5 g/L in 50 mL LOD medium with 40 mM MOPS and incubated at 65 °C with shaking at 150 rpm. Cell growth on cellobiose was measured by optical density (OD) at 680 nm using a Jenway Genova spectrophotometer. To measure growth on oat spelts and birchwood xylans, both the sub-culture and the initial culture were performed in LOD medium with 5 g/L oat spelts and birchwood xylans. Colony-forming units (CFU) were measured by plating cells on LOC medium with 5 g/L maltose and incubating at 65 °C for 3 days. We previously reported plating efficiencies for LOD medium [29, 39] and recently showed that the plating efficiencies for the LOD and LOC mediums are the same.

## Additional file

10.1186/s13068-016-0588-9 List of primers used in this study. **Fig. S1.** Verification of the stable presence of expression vectors for Acel_0180 and Acel_0372 in *C. bescii* transformants. **Fig. S2.** Sequence alignments of CBM family 2. **Fig. S3.** Relative enzymatic activity of the extracellular fraction of *C. bescii* strains on oat spelts and birchwood xylans after 1 h incubation at 75°C. **Fig. S4.** Relative enzymatic activity of the extracellular fraction of *C. bescii* strains on oat spelts and birchwood xylans after 1 h (A) or 12 h (B) incubation at 65°C. **Fig. S5.** Sequence alignments of CBM family 3. **Fig. S6.** Sequence alignments of GH10s highlighting the catalytic residues (*) and xylan binding site (bold). **Fig. S7.** Comparison of the homology models of the two GH10s from Acel_0180 (A, C) and Acel_0372 (B, D) using PDB structures 3WUF and 2CNC as templates, respectively. **Fig. S8.** Viable cell numbers after 12 h (A) and 24 h (B) cultivation on xylan substrates. **Fig. S9.** Photographs of 125-mL serum bottles containing 25 mL LOD medium with 0.5 % (w/v) birchwood xylan after 24 h cultivation with *C. bescii* strains. **Fig. S10.** Scheme of plasmid pSKW10 construction.

## Abbreviations

*C. bescii*: *Caldicellulosiruptor bescii*
*A. cellulolyticus*: *Acidothermus cellulolyticus*
CBM: carbohydrate-binding domain
GH: glycoside hydrolase
CAZy: carbohydrate-active enzymes
CMC: carboxymethylcellulose
5-FOA: 5-fluoroorotic acid
LOD: low osmolarity defined
LB broth: Luria–Bertani broth
ECP: extracellular protein
BSA: bovine serum albumin
DNS: dinitrosalicylic acid
SDS: sodium lauryl sulfate
OD: optical density
MOPS: 3-(*N*-morpholino)propane sulfonic acid
MES: 2-(*N*-morpholino)ethanesulfonic acid
