# Supplementary material for: Heterologous expression of family 10 xylanases from Acidothermus cellulolyticus enhances the exoproteome of Caldicellulosiruptor bescii and growth on xylan substrates
Source: Biotechnol Biofuels. 2016 Aug 22;9(1):176. doi: 10.1186/s13068-016-0588-9 (PMC4994175; doi:10.1186/s13068-016-0588-9)
Supplement: Supplementary file 1 — 10.1186/s13068-016-0588-9 List of primers used in this study. Fig. S1. Verification of the stable presence of expression vectors for Acel_0180 and Acel_0372 in C. bescii transformants. Fig. S2. Sequence alignments of CBM family 2. Fig. S3. Relative enzymatic activity of the extracellular fraction of C. bescii strains on oat spelts and birchwood xylans after 1 h incubation at 75°C. Fig. S4. Relative enzymatic activity of the extracellular fraction of C. bescii strains on oat spelts and birchwood xylans after 1 h (A) or 12 h (B) incubation at 65°C. Fig. S5. Sequence alignments of CBM family 3. Fig. S6. Sequence alignments of GH10s highlighting the catalytic residues (*) and xylan binding site (bold). Fig. S7. Comparison of the homology models of the two GH10s from Acel_0180 (A, C) and Acel_0372 (B, D) using PDB structures 3WUF and 2CNC as templates, respectively. Fig. S8. Viable cell numbers after 12 h (A) and 24 h (B) cultivation on xylan substrates. Fig. S9. Photographs of 125-mL serum bottles containing 25 mL LOD medium with 0.5 % (w/v) birchwood xylan after 24 h cultivation with C. bescii strains. Fig. S10. Scheme of plasmid pSKW10 construction. [file 13068_2016_588_MOESM1_ESM.docx]

**Supplementary data**

Table S1. List of primers used in this study. The italicized sequences indicate the recognition sites of the corresponding restriction enzymes

| Name | Sequence (5’ → 3’) | Restriction enzyme | Description |
| --- | --- | --- | --- |
| DC699 | ACTATCT*GTGCAC*AAACGAACCAGCCCTAACCTCT | ApaLI | To construct pDCW213 & 214 |
| DC700 | AGA*CCTAGG*CATCACCATCACCATCACTAATAAT | AvrII |  |
| DC701 | ACTATCT*GTGCAC*TCCACCTTGAAACAGGGGGCGGA | ApaLI | To construct pDCW213 |
| DC702 | AGA*CCTAGG*GGAGGTGGTGCAGGTAAAGGT | AvrII |  |
| DC703 | ACTATCT*GTGCAC*AATCCGCCGTACCACCCGC | ApaLI | To construct pDCW214 |
| DC704 | AGA*CCTAGG*TCGGCGCAGGGCGTGATGA | AvrII |  |
| DC460 | AGAGAG*CGATCG*ACAGTTTGATTACAGTTTAGTCAGAGCT | PvuI | To construct pSKW10 |
| DC461 | AGAAGAAG*GCGGCCGC*TTGGTTCCTTAAATCTAAGAGGTATGA | NotI |  |
| DC481 | AGACTC*CGATCG*ATTCCCATGAGCCCACGAACAGT | PvuI | To construct pSKW10 |
| DC482 | AGAAGAAG*GCGGCCGC*TCTGACGCTCAGTGGAACGAA | NotI |  |
| DC560 | ACT*GGATCC*ATGAAGCGTTACAGAAGAATTATTGCCA | BamHI | To construct pSKW11 |
| DC461 | AGAAGAAG*GCGGCCGC*TTGGTTCCTTAAATCTAAGAGGTATGA | NotI |  |
| DC464 | ACT*GGATCC*CTCACCAAACCTCCTTGTATGAT | BamHI | To construct pSKW11 |
| DC482 | AGAAGAAG*GCGGCCGC*TCTGACGCTCAGTGGAACGAA | NotI |  |
| DC460 | AGAGAG*CGATCG*ACAGTTTGATTACAGTTTAGTCAGAGCT | PvuI | To confirm transformants |
| DC228 | ATCATCCCCTTTTGCTGATG | - |  |


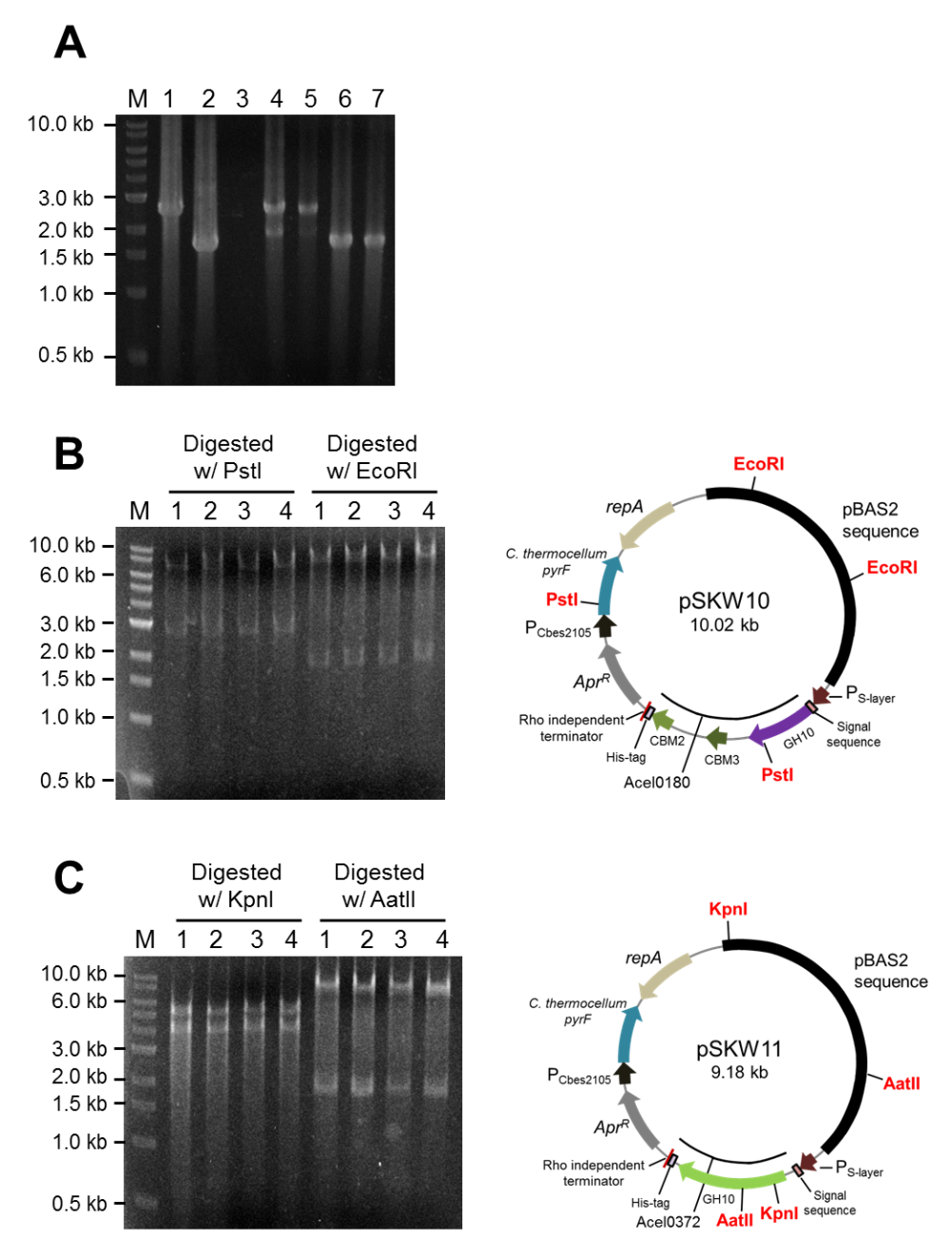


**Figure S1. Verification of the stable presence of expression vectors for Acel_0180 and Acel_0372 in *C. bescii* transformants.** (A) Gel showing the 2.4 and 1.6 kb PCR products using plasmid-specific primers DC460 and DC228. 1: plasmid pSKW10; 2: plasmid pSKW11; 3: negative control JWCB52; 4 and 5: JWCB74 (JWCB52 *+* pSKW10); 6 and 7: JWCB75 (JWCB52 *+* pSKW11). (B) Restriction analysis of pSKW10 plasmid DNA before and after transformation of *C. bescii* and back-transformation to *E. coli*. 1: pSKW10 plasmid DNA isolated from *E. coli* DH5α, digested with either PstI (7.2 kb and 2.9 kb cleavage products), or with EcoRI (8.2 kb and 1.9 kb cleavage products), 2, 3, and 4 : plasmid DNA isolated from three biologically independent *E. coli* DH5α back-transformants using total DNA isolated form *C. bescii* transformants, digested with either PstI or EcoRI (C) Restriction analysis of pSKW11 plasmid DNA before and after transformation of *C. bescii* and back-transformation to *E. coli*. 1: pSKW11 plasmid DNA isolated from *E. coli* DH5α, digested with either KpnI (5.2 kb and 4.0 kb cleavage products), or with AatII (7.4 kb and 1.8 kb cleavage products), 2, 3, and 4 : plasmid DNA isolated from three biologically independent *E. coli* DH5α back-transformants using total DNA isolated form *C. bescii* transformants, digested with either KpnI or AatII.

**
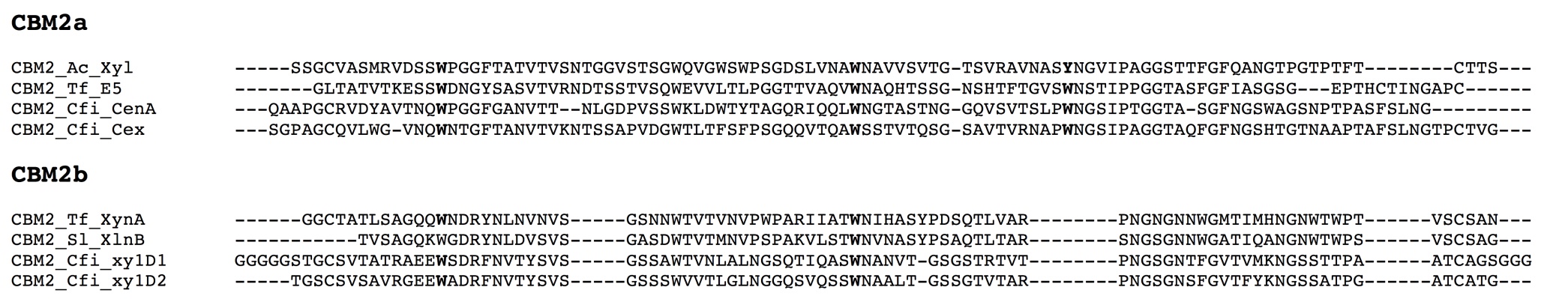
**

**Figure S2. Sequence alignments of CBM family 2.** The conserved residues determining the type of the CBMs are shown in bold. The origin of the CBMs is as follows: CBM2_Ac_Xyl, *A. cellulolyticus* Acel_0180, CMB2_Tf_E5, *T. fusca* endoglucanase E5, CBM2_Cfi_CenA, *C. fimi* endoglucanase A, CfiCex, *C. fimi* xylanase A, CBM2_Tf_XynA, *T. fusca xylanase* A*, CBM2_Sl_XlnB, S. lividans* xylanase B, *CBM2_Cfi_xylD, C. fimi xylanase D.*


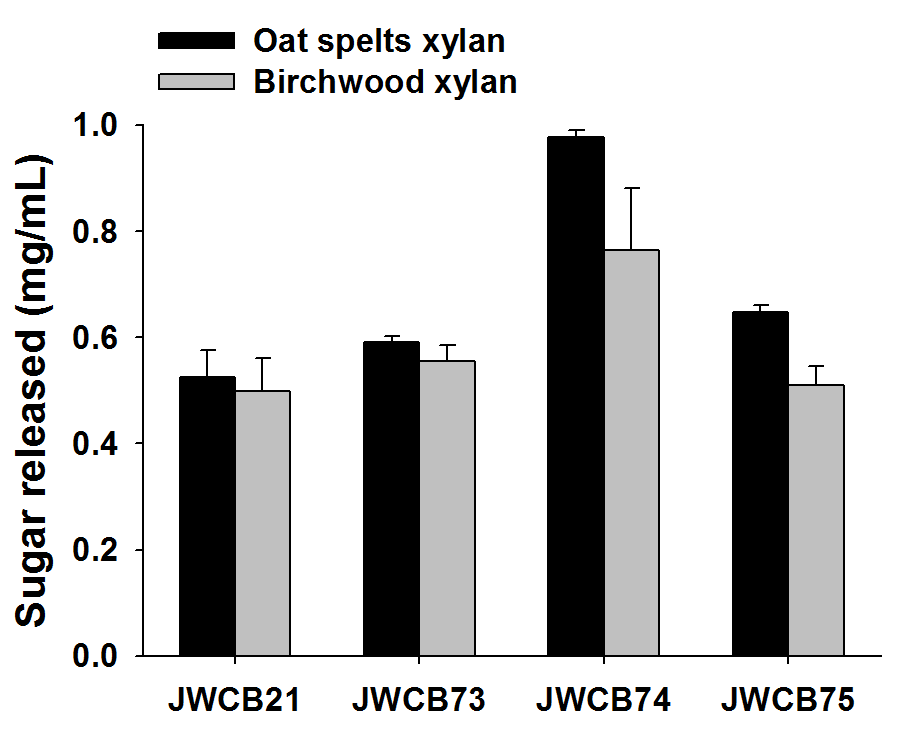


**Figure S3. Relative enzymatic activity of the extracellular fraction of *C. bescii* strains on oat spelts and birchwood xylans after 1 h incubation at 75°C.** Activity of extracellular protein (25 µg/mL concentrated protein) on oat spelts and birchwood xylans was measured in triplicate. JWCB21, the parent strain used in these experiments (see Table 2 for genotype details); JWCB73, the E1 expression strain; JWCB74, the E1 expression strain containing Acel_0180; JWCB75, the E1 expression strain containing Acel_0372.


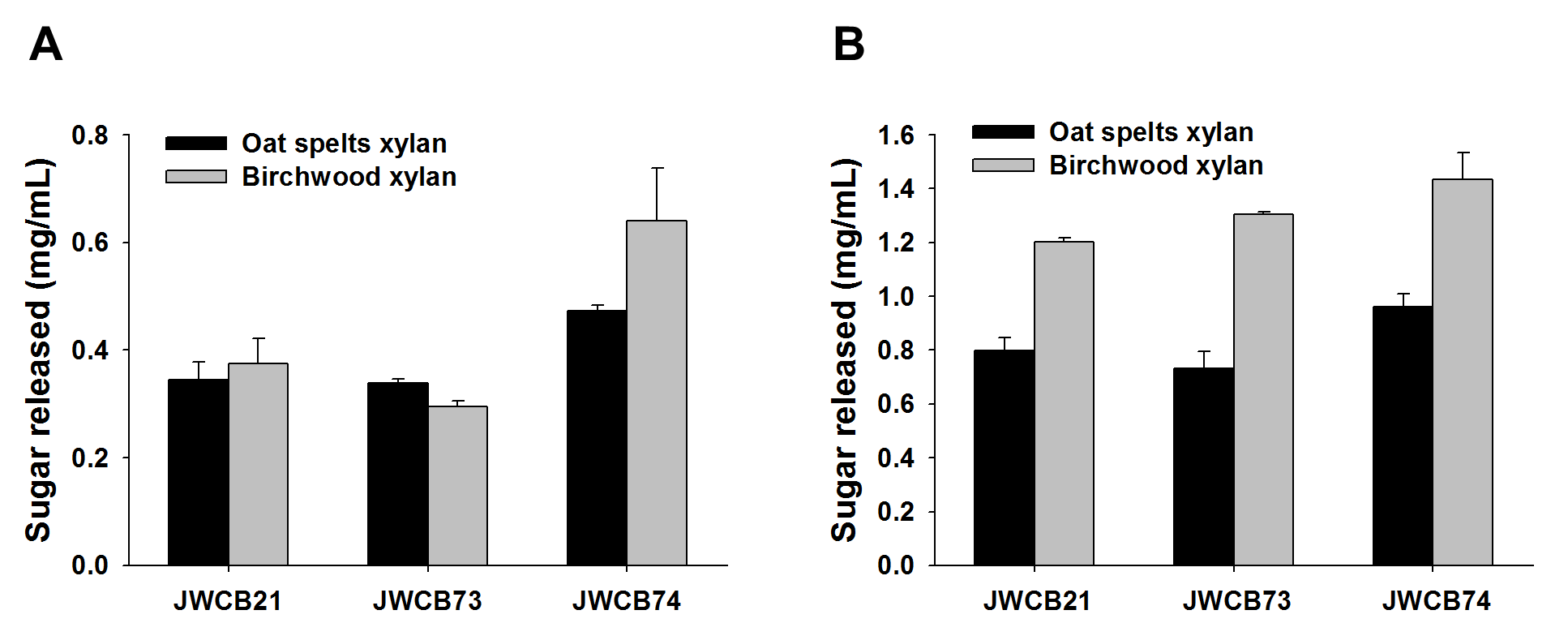


**Figure S4. Relative enzymatic activity of the extracellular fraction of *C. bescii* strains on oat spelts and birchwood xylans after 1 h (A) or 12 h (B) incubation at 65°C.** Activity of extracellular protein (25 µg/mL concentrated protein) on oat spelts and birchwood xylans was measured in triplicate. JWCB21, the parent strain used in these experiments (see Table 2 for genotype details); JWCB73, the E1 expression strain; JWCB74, the E1 expression strain containing Acel_0180.


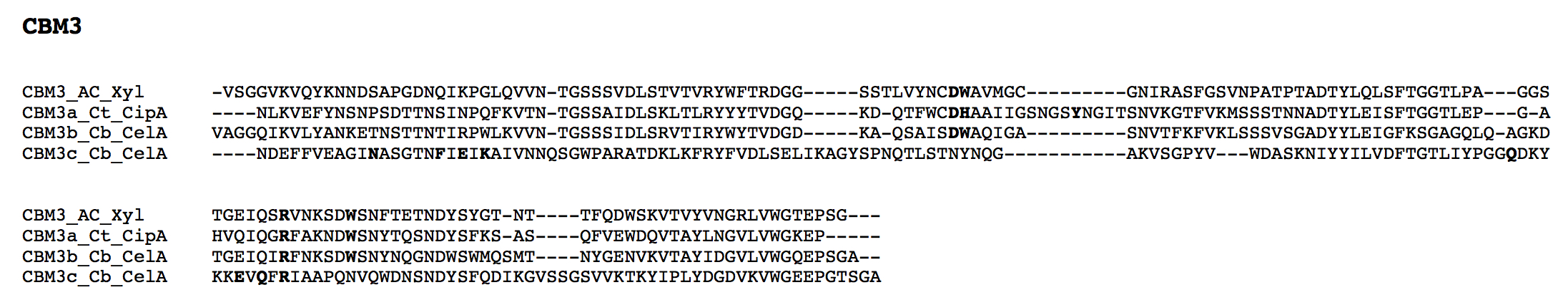


**Figure S5. Sequence alignments of CBM family 3.** The conserved residues determining the type of the CBMs are shown in bold. The origin of the CBMs is as follows: CBM3_Ac_Xyl, *A. cellulolyticus* Acel_0180, CMB3_Ct_CipA, *C. thermocellum* CipA*,* CBM3b_Cb_CelA, *C. bescii CelA*, CBM3c_Cb_CelA, *C. bescii CelA*


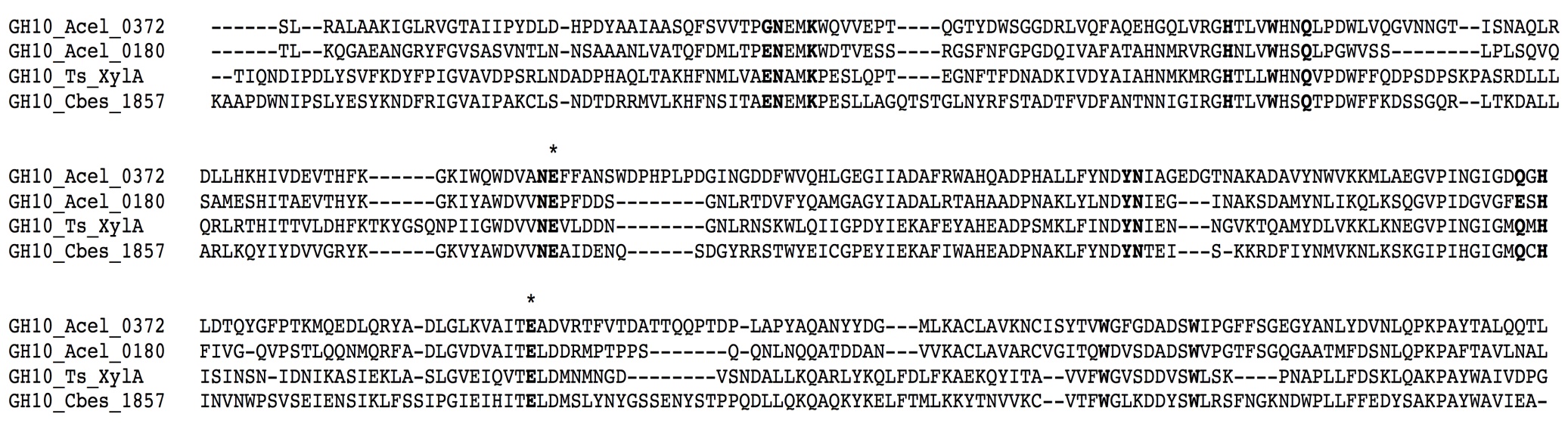


**Figure S6. Sequence alignments of GH10s highlighting the catalytic residues (*) and xylan binding site (bold).** The origin of the GH10s is as follows: GH10_Acel_0372, *A. cellulolyticus* Acel_0372, GH10_Acel_0180, *A. cellulolyticus* Acel_0180, GH10_Ts_XylA, *T. saccharolyticuym* xylanase A, GH10_Cbes_1857, *C. bescii* Cbes_1857*.*


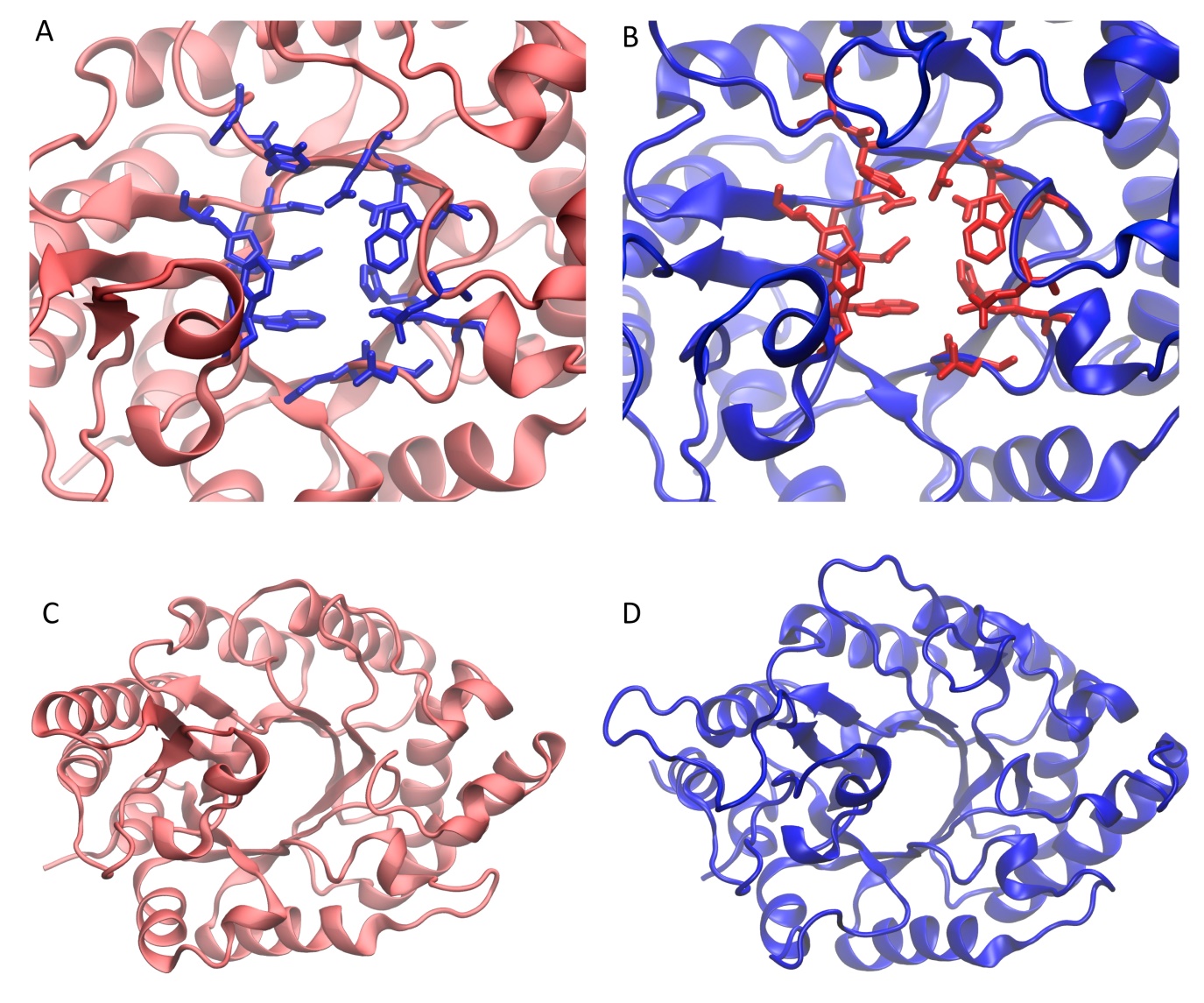


**Figure S7. Comparison of the homology models of the two GH10s from Acel_0180 (A, C) and Acel_0372 (B, D) using PDB structures 3WUF and 2CNC as templates, respectively.** A and B highlight the catalytic sites and C and D show the overall structure of the two GH10s.


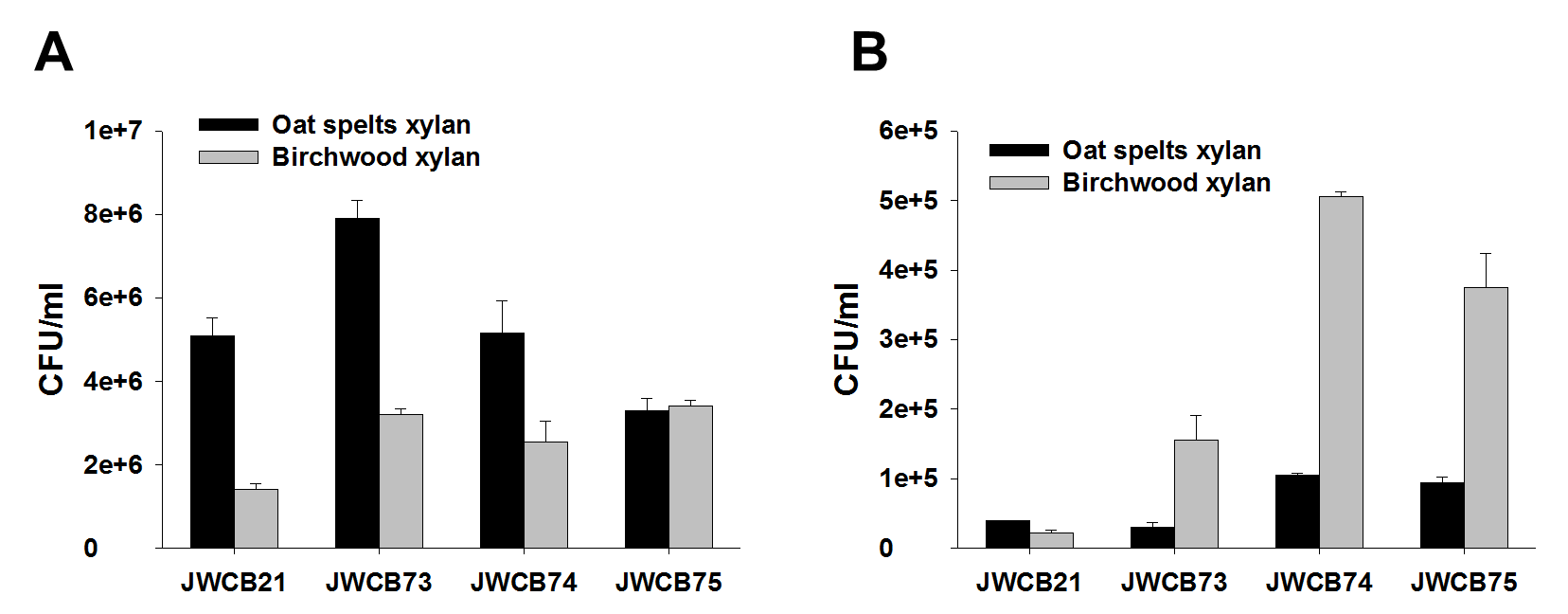


**Figure S8. Viable cell numbers after 12 h (A) and 24 h (B) cultivation on xylan substrates.**  JWCB21, the parent strain used in these experiments (see Table 2 for genotype details); JWCB73, the E1 expression strain; JWCB74, the E1 expression strain containing Acel_0180; JWCB75, the E1 expression strain containing Acel_0372.


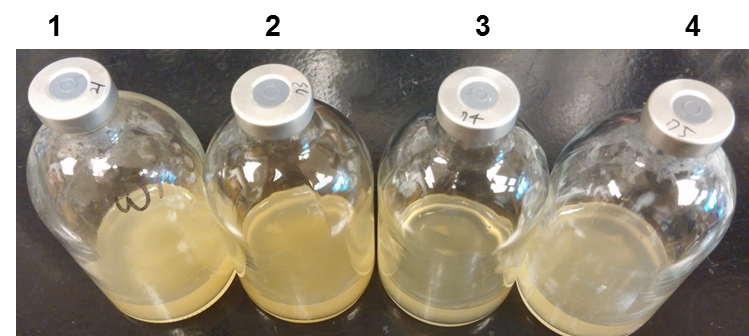


**Figure S9. Photographs of 125-mL serum bottles containing 25 mL LOD medium with 0.5 % (w/v) birchwood xylan after 24 h cultivation with *C. bescii* strains.** 1, JWCB21 (parental strain); 2, JWCB73 (E1 expressing strain); 3, JWCB74 (E1 + Acel_0180 expressing strain); 4, JWCB75 (E1 + Acel_0372 expressing strain).


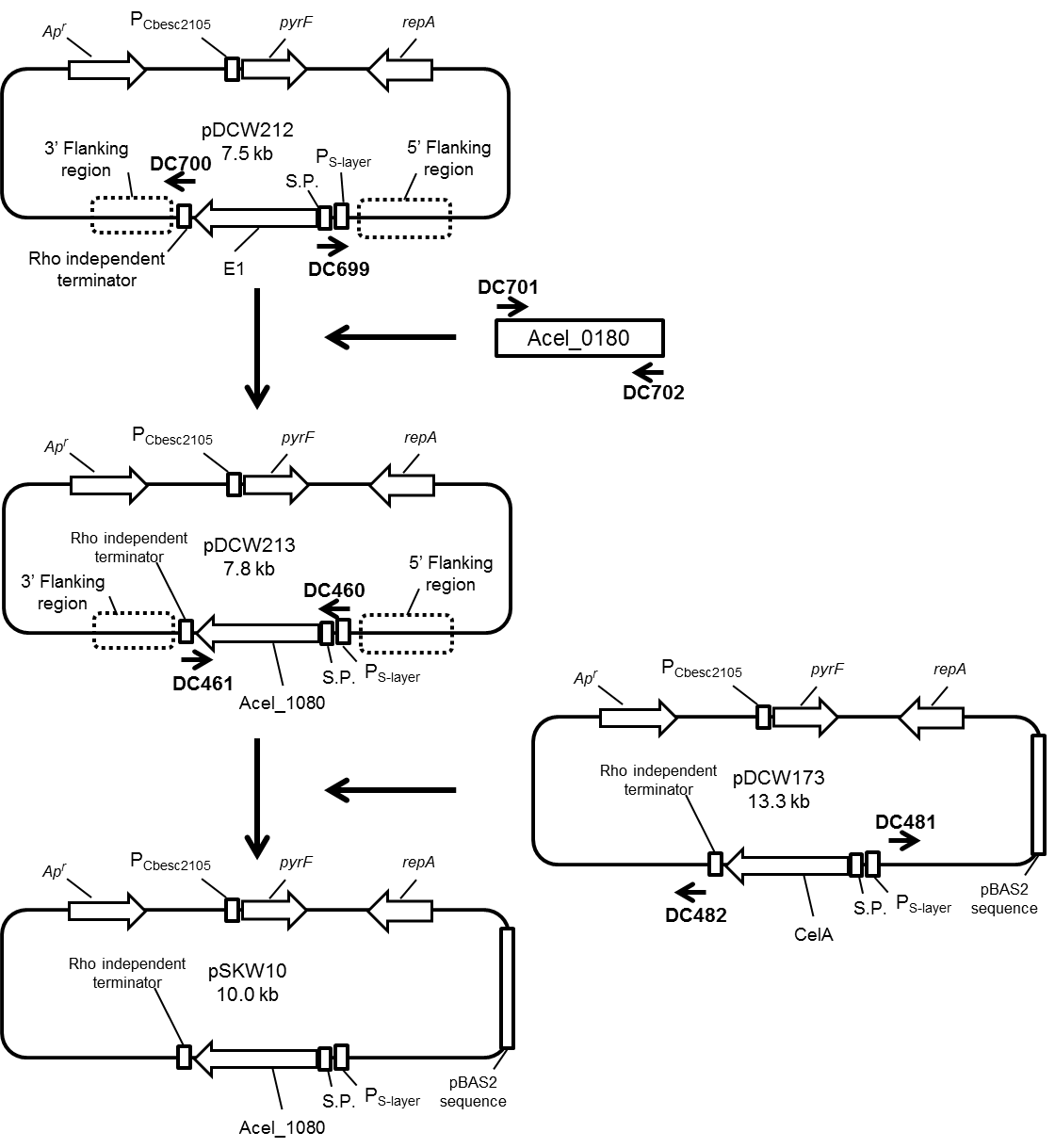


**Figure S10. Diagram of plasmid pSKW10 construction.**
